# Supplementary material for: Program evaluation of a wilderness experience for adolescents facing cancer: A time in nature to heal, connect and find strength
Source: PLoS One. 2023 Oct 3;18(10):e0291856. doi: 10.1371/journal.pone.0291856 (PMC10547176; doi:10.1371/journal.pone.0291856)
Supplement: S1 Appendix — (DOCX) [file pone.0291856.s001.docx]

**S1 Appendix.** Table 1-3 Provides more detailed information regarding program activities (Table 1), staff qualifications and roles (Table 2), and supplementary quotes (Table 3)

| **Appendix Table 1: Daily Goals, Events implemented and Lessons Learned** | | | |
| --- | --- | --- | --- |
| *Day* | *Overarching Daily Goals* | *Events as actually implemented* | *Discussion and Lessons Learned* |
| *Day 1* | *Psychomotor & cognitive:*  -Wilderness skills (introduction)  -Physical movement (yoga)  *Psychological experiences:*  Peer introductions, group orientation, pet therapy, music therapy, self reflection, psychological resilience tools introduced  *SYATS:* fresh food at base camps, easing in  *Medical:* check- in, meds | -Participant and staff arrival.  -Introductions  -Registration for medication and medical record for each teen and staff.  -Gear/clothing organization  -Beginning group work.  -Pet therapy  Teambuilding through camping  -Wilderness and backpacking education  -Music with yoga  -Staff training  -Closing circle: debrief, next day prep, staff meeting | -Intro: include more ice breaker activities on Day 1 to help break down social barriers.  -Consider pre-arrival wilderness education.  -Staff meeting at end of day crucial for staff check-in, further creating staff culture, reviewing teens needs and reinforcing key program principles.  -Tasks (outfitting with clothing and gear etc.) was labor intensive.  -Conclusion: More team building, assign designated staff for outfitting. |
| *Day 2* | *Psychomotor & cognitive:*  -Wilderness skills (cont. intro to: hiking, pack, bathroom hygiene, camp set up chores, and safety)  -Physical movement (hike, stretching)  *Psychological experiences:*  psychological resilience tools (gratitude, journaling, meditation), Self-efficacy through physical accomplishment of new skills | -AM check in, meditation, camp break down, revisited group process and goals, stretching.  -Education: Hiking education, boots and blisters, packing & pack fitting, going to the bathroom in the woods, leadership introduction.  -Pet Therapy.  -Hiked indicated route.  -Intro to camp chores and basic needs upon arrival to camp.  -Closing circle: debrief, next day prep, staff meeting. | -Teens were eager to journal after hike.  -Wilderness education took significant amount of time even when spread out through the first 2 days. This is a good day to learn about basic needs of self in wilderness. -Challenged to find the balance between teaching and down-time. |
| *Day 3* | *Psychomotor& cognitive:*  -Wilderness skills (building competency on existing skills, intro to outdoor cooking, basic needs.  -Physical movement (swimming, stretching, hiking to and from campsite)  *Psychological experiences:*  build on resiliency skills, self-confidence increasing through competency of skills, introducing mindfulness, develop peer connectedness and belonging through share experience and having fun  *SYATS*: Leadership training, caring for others inclusion, belonging | *Rest day chosen instead of day hike.  -Camp experience: Teen independence over meals,  -Camp chores  and camp education continued.  - Ranger met with teens to give wilderness education and leave no trace lesson.  -Journaling, mindfulness, stretching  -Facilitation- Teambuilding  -Free time: swimming, bonding  -Leadership prep  -Closing circle: debrief, next day prep, staff meeting | -Planned hike was replace with rest day responsive to participant need. Scheduling flexibility is key.  -Staff need training on camping gear they are unfamiliar with. |
| *Day 4* | *Psychomotor& cognitive:*  -Wilderness skills (Re-naturalizing our impact, self-care check in, camping routine/time management  -Physical movement (stretching, hiking, increased weight in backpacks)  *Psychological experiences:*  Teambuilding  Resiliency skills, self-efficacy through incremental challenges, peer negotiation, pet therapy,  *SYATS:* Identity curriculum, leadership training, caring for others | -Am check in, meals self-directed,  -Campsite break down, navigating time management, reinforced self-care, routine forming for independent directed tasks regarding camping and backpacking.  -Stretched.  -Hiked indicated route.  -Free time: swimming  -Facilitated discussion with participant #3 on trail. Pet therapy during hike.  -Leadership prep.  -Closing circle: debrief, next day prep, staff meeting | -SYATS curriculum is crucial for self-reflection. Deepens relationships and self-awareness specific to these teens’ life experiences/identify.  -Group formation process was on track. Group and individual challenges surfaced which allowed future growth on days 5-8.  (One year after trip, Participant #3 talked about being able to better communicate emotions to parents). |
| *Day 5* | *Psychomotor & cognitive:*  -Wilderness skills (becoming routine)  -Physical (stretching, hiking)  *Psychological experiences:*  psychological resilience skills, peer relations, emotional regulation and vulnerability,  Teambuilding (tent pole),  *SYATS*: Impact, communication, leadership training, caring for others, fresh food at base camps | -AM check in w/ breakfast,  -campsite break down/ chores, -Stretching  -Facilitated activities: teambuilding- tent pole activity- group dynamics brought to the surface and facilitated  -Journaling, group debrief, teens time management struggle around basic needs and natural consequences.  -Facilitated conversation with participant #7 regarding group role, having hard conversations and giving feedback  -Medical: allergies, nosebleed  -2 hiking groups occurred due to nosebleed.  -Hiked indicated route, with groups reconnected half-way through. (Longest hike yet.)  - Two teens spontaneously led dinner and advocated for others to be responsible for clean up.  -Camp fire with music.  -Closing circle: debrief, next day prep, staff meeting | -Teens struggled with cohesion, time management and hiking prep. Facilitated activity to bring tension to the surface, reflection time and debrief crucial to group shifting to deeper belonging and trust.  -Celebratory atmosphere noted in late afternoon evening.  -Spontaneous singing, laughing and joking noted.  -Increased level of trust resulting in greater emotional risk taking among the group.  -Noted possibility of previous communication struggle as a result of not wanting to be perceived as suffering through honest feedback. Wondered if this population may be more sensitive to perception of causing suffering in others among shared cancer experience.  -Though the protocol does not include dividing the participants into separate hiking groups this was done due to a minor medical need. Participant interviews showed this was acceptable and provided its own challenge and sense of accomplishment. Another example of how experiential education is flexible to what the environment and situation present. |
| *Day 6* | *Psychomotor & cognitive:*  -Wilderness skills (selfcare, environmental impact)  -Physical (relaxing)  *Psychological experiences:*  psychological resilience skills-included solo time, peer relations, mindfulness meditation, teambuilding  *SYATS*: summit day/CA metaphor discussion, summit day identity reflection prep, resiliency skill modelling and implementation, looking outward -acknowledging funders, donors and support systems, receiving support: Donors letters to teens, leadership training, caring for others, fresh food at base camps | Rest day  -teens slept in, independent breakfast.  -Revisited tent pole, Swimming, laundry.  -Facilitated conversation around summit day anticipation and personal reflections on moving forward  -Mindfulness meditation (raisin)  -Solo time, journaling  -Summit day packing prep  -Staff made dinner to allow for more free time  -Camp fire, music  -gratitude cards, looking outward  -donor letters to teens  -Leadership prep  -Closing circle: debrief, next day prep, staff meeting | -This day was invaluable. It provided time for teens to rest and reflect. It also allowed for longer meditation practice to be introduced. There was an ease now that teens had worked through some of the group tension. It was also valuable to have them do camp chores-(laundry). |
| *Day 7* | *Psychomotor& cognitive:*  -Wilderness skills (Summit/cliff awareness )  -Physical (stretching, hiking)  *Psychological experiences:*  psychological resilience skills- included silent hiking, belonging and group support, increases self-efficacy and perceived capacity through large challenge  *SYATS*: Summit occurs at the highest point the intact group can achieve  Together, may not be the actual indicated summit. Challenge day, Rock exercise, What’s next, Individual photos at summit | Summit day,  -Early rising, Am snack,  Stretching, meditation  -staff supported campsite break down.  -Hiked indicated route  -stop for b-fast on trail,  -Medical: one teen noted allergies/sinus symptoms. Presented a staff logistical and teen leadership challenge.  Remaining teen lead worked through anxiety about leading without colead. MD and RN delayed with one teen while meds given and symptoms resolved.  -stopped for snack, swam in creek, teen, MD and RN rejoined teens and staff, Rock exercise introduced.  -silent hike portion  -group dynamic awareness  -meet up with pack horses at next campsite.  -Teen led check-in on the trail.  -Near summit: Rock debrief  -whole group summited,  Individual photos, group photo.  -Return hike to pre-established campsite, camp chores  -dinner, surprise dessert  -Closing circle: summit day debrief. Quiet time with guided journal prompts, next day prep, teens stayed up with staff and played some circle games  -staff meeting | -Rock exercise was very powerful for all teens.  -Group leader exercise was successful.  -Silent hiking valuable.  -Teens did quiet journaling as part of the summit debrief instead of solo time.  -In the future, an extra medical personal at base camp could be helpful in case one person had to hike down or hike out.  -Having volunteers prepack and establish campsite was valuable.  -Utilization of pack horses valuable. |
| *Day 8* | *Psychomotor& cognitive:*  -Wilderness skills (routine)  -Physical (hiking)  *Psychological experiences:*  psychological resilience skills- included silent hiking, strengthen self-concept, transference to real life  *SYATS*: personal themes, letters to self, letters to family, looking outward acknowledging funders, donors and support systems,  fresh food at base camps | (No sunrise hike)  -forecast indicated rain, decision made by safety officer and medical director to pack up and get hiking.  -leadership prep done in AM this day  -Bars for breakfast.  -Camp breakdown.  - Hiked indicated route: backcountry  Facilitated discussion on personal themes and next steps, quiet hike contemplating personal themes.  -one teen mis-stepped and tweaked ankle, relieved of pack completed hike.  -hike completion celebration with snack surprise  -exited interviews for qualitative research started  -gear return  -Campfire: gratitude circle.  -Closing circle: debrief, next day prep. No staff meeting | -Due to rain forecast and descending hike it was determined that we needed an early start for the safest experience. Teens packed an energy bar and their immediate bags and hike started early. Instead of a group debrief they had quiet hike time and debrief during hiking breaks. Possibly some value lost in not having time to reflect and celebrate previous day summit. Again, flexibility key.  -Volunteers aiding in returning large amount of gear down the mountain.  -The surprise pizza delivery was a success and brought smiles to everyone.  -Positive circle was done in the evening around the campfire.  -Helpful to start gear return early.  -Day 8 protocol outlined two schedules allowing for the unknown needs of the group |
|  |  |  |  |
| *Day 9* | *Psychological experiences:*  psychological resilience skills, teambuilding activity, strengthen self concept, transference to real life,  *SYATS*: looking outward, looking forward (make breakfast for staff, letters to family, acknowledging funders, donors and support systems)  fresh food at base camps  *Medical: check out medications* | -AM circle, meditation, breakfast (teens did not cook breakfast for staff)  -quantitative data collected  -qualitative data interviews completed  -letters to self  -completed gratitude notes for physician, sponsors and donors  -to go lunches made  -final camp breakdown, last gear checked in,  -Closing circle  -staff and teens departed | -Teens cooking breakfast for staff did not happen due to time and energy shortage however the skill of “looking outward” was achieved through writing the gratitude letters.  -Letters to physicians were written instead of family letters, as some teens indicated this was their preference. |

| **Appendix Table 2:** Staff qualifications | | |  |
| --- | --- | --- | --- |
| ***Category*** | | ***Role/Notes*** |  |
| Contact with teens | |  |  |
| Photographer (Male) | | Documented trip for outreach, program development, fundraising, and reliving experience for participants. |  |
| 2 Emergency Physicians (Male) | | Tracked health issues and delivered prescribed medications |  |
| Wilderness/outdoor adventure facilitator and mother of a child who had cancer (Female) | | Co-facilitator |  |
| Nurse, researcher, wilderness/outdoors experience (MJ, Male) | | Volunteer researcher (PhD), shadowing program to learn and be inspired for establishing a similar program in Sweden |  |
| Oncology Registered Nurse (RN), outdoor experience (Male) | | Chaperone, administered medication |  |
| Personal trainer, Physiotherapist, physical fitness expertise (Female) | | Lead stretching and yoga sessions |  |
| Wilderness Adventure Facilitator, Intensive Care unit (ICU) RN (HRO, Female) | | Conceived of program, developed protocol and lead the trip. |  |
| RN (Female) | | Chaperone, administered medication |  |
| On-site or nearby with or without minimal contact with teens | |  |  |
| 2 pediatric oncologists-on call | |  |  |
| ICU RN (Female) | | Chaperone, administered medication |  |
| ICU RN, horse handler | | transported gear |  |
| 6 volunteers with military search and rescue and coordination experience | | Served as back-up safety and gear |  |
| Teacher | | Chaperoned Seattle Teens on flight |  |
| Psycho-oncology researcher and mother of cancer survivor (EAL, Female) | | Provided guidance for program evaluation |  |
| On-call personnel | |  |  |
| 2 licensed psychotherapists | | Served as backup for psychosocial issues |  |
| **APPENDIX, TABLE 3: Supplementary quotes from participants, described by theme.** | | | |
| **Topic** | **Quotes** | | |
| **Acceptance of SYATS program-teambuilding** | *Even though the pole [exercise] was frustrating, I think that one brought us together the most, so I think I liked that one the best.” (participant 4.)* | | |
| **Teambuilding** | *“It’s a troubling exercise. It took us a while. Eventually we got it, and I feel like that exercise was trying to teach us to trust in one another…. that we shouldn’t just think we’re all on our own, and that we have someone to help us, and we’re a team and we can trust one another. I felt there could’ve been more of those teambuilding exercises because they did help us and plus they were just a lot of fun…” (participant 1)* | | |
| **Teambuilding** | *“At first I felt like the teambuilding was a bit much, but I do like it and think we should’ve done a couple more because we definitely had frustrations within the group, but those frustrations built a stronger dynamic because if we didn’t confront the problems making us not work together, that piece that was holding us apart would’ve stayed there the entire week and wouldn’t get any better.” (participant 8)* | | |
| **Health** | *“I got it the night before, but I started feeling better and then worse again, but way worse. I kind of got a headache, sinuses were clogged, sore throat. I was just feeling weak overall. Stomachache. But yeah after like I’d say less than an hour, everything but the sinuses went away. I’m still feeling the sinuses right now.” (participant 1)* | | |
| **Acceptability** | In the interviews, several of the teenagers expressed that they were initially uncertain they had the mental and physical capacity to complete the program.  *“I thought it’d be a lot harder than it was. After that first day, I think it settled in that you’re doing this and you can’t get out of it now. You just kind of have to do it to your best ability, and I think I did that.” (participant 4)* | | |
