# Supplementary material for: Program evaluation of a wilderness experience for adolescents facing cancer: A time in nature to heal, connect and find strength
Source: PLoS One. 2023 Oct 3;18(10):e0291856. doi: 10.1371/journal.pone.0291856 (PMC10547176; doi:10.1371/journal.pone.0291856)
Supplement: S2 Appendix — (PDF) [file pone.0291856.s002.pdf]

Consolidated criteria for reporting qualitative studies (COREQ): 32-item checklist
Manuscript title: *Program evaluation of a wilderness experience for adolescents facing cancer: A time in nature to heal, connect and find strength*

No. Item	Guide questions/description	Reported in heading
**Domain 1: Research team and reflexivity**		
Personal Characteristics		
1. Interviewer/Participant observer	Which author/s conducted the interview or focus group?	Heading:MATERIAL AND METHODS -- *Qualitative data*
2. Credentials	What were the researcher's credentials? E.g. PhD, MD	Not in line with PLOS requirements to state credentials, but is stated for the interviewer in table 2
3. Occupation	What was their occupation at the time of the study?	Table 2
4. Gender	Was the researcher male or female?	Both: Heading:MATERIAL AND METHODS -- *Qualitative data*
5. Experience and training	What experience or training did the researcher have?	All researchers have both training and previous Qualitative research experience.
Relationship with participants		
6. Relationship established	Was a relationship established prior to study commencement?	No, not for interviewer/researcher prior to program start.<br>Program director had contact during recruitment process<br><br>.
7. Participant knowledge of the interviewer	What did the participants know about the researcher? e.g. personal goals, reasons for doing the research	The overall purpose of the study was clearly briefed for the participants as described in<br>MATERIAL AND METHODS sections: *SYATS Program --- Qualitative data ---- Human Subjects Ethics and Consent Process*
8. Interviewer characteristics	What characteristics were reported about the inter viewer/facilitator? e.g. Bias, assumptions, reasons and interests in the research topic	The Interviewers/Participant observer has stake on the program being studied besided having an interest in shadowing program for research purposes, as well as for inspiration to start similar program in Sweden – See

		table 2

**Domain 2: study design**		
Theoretical framework		
9. Methodological orientation and Theory	What methodological orientation was stated to underpin the study? e.g. grounded theory, discourse analysis, ethnography, phenomenology, content analysis	The mixed methods design has a pragmatic approach with no a'priori theorethical underpinning. Qualitative content analysis was performed on interview data: MATERIAL AND METHODS sections: *Description of study design --- Data analysis*
Participant selection		
10. Sampling	How were participants selected? e.g. purposive, convenience, consecutive, snowball	MATERIAL AND METHODS sections: *SYATS Program --- Human Subjects Ethics and Consent Process*<br><br>A combination of purposive and convenience sampling: reaching out to key persons with access to the target group
11. Method of approach	How were participants approached? e.g. face-to-face, telephone, mail, email	Face-to face, by tlephone and email. MATERIAL AND METHODS sections: *SYATS Program --- Human Subjects Ethics and Consent Process*
12. Sample size	How many participants were in the study?	Table 1, and under heading RESULTS --- *Feasibility data, Aim 1, quantitative and qualitative findings*
13. Non-participation	How many people refused to participate or dropped out? Reasons?	None – A part of the feasibility data, shown

		in RESULTS --- *Feasibility data, Aim 1, quantitative and qualitative findings*
Setting		
14. Setting of data collection	Where was the data collected? e.g. home, clinic, workplace	All qualitative and quantitative data was collected in the field while program was ongoing. Three month follow up data was collected by emailed or regular mail<br>Shown in: MATERIAL AND METHODS sections: *Description of study design* --- *Quantitative data* --- *Qualitative data*<br>.
15. Presence of non-participants	Was anyone else present besides the participants and researchers?	Not in the setting where interview data was collected. But as this is an evaluation of a wilderness program, a number of other people was included in the observation of interaction (Table 2
16. Description of sample	What are the important characteristics of the sample? e.g. demographic data, date	Shown in table 1
Data collection		
17. Interview guide	Were questions, prompts, guides provided by the authors? Was it pilot tested?	As described in Heading: MATERIAL AND METHODS -- *Qualitative data*<br>*Both informal interviews, and exit interviews were performed in line with the design of having a particpant observer.*<br>The exit interview guide is attached as Supplement S3

18. Repeat interviews	Were repeat interviews carried out? If yes, how many?	Yes, shorter ad hoc interviews was made multiple times during the program with participants, exit interviews only once. As described in Heading: MATERIAL AND METHODS -- *Qualitative data*
19. Audio/visual recording	Did the research use audio or visual recording to collect the data?	Audio, field diary and paper surveys. As described in Heading: MATERIAL AND METHODS -- *Qualitative data* --- *quantitative data*
20. Field notes	Were field notes made during and/or after the interview or focus group?	Yes, observations continuosly documented in field diary, as described in Heading: MATERIAL AND METHODS -- *Qualitative data*
21. Duration	What was the duration of the interviews or focus group?	Described in Heading: MATERIAL AND METHODS -- *Qualitative data*
22. Data saturation	Was data saturation discussed?	As being a program evaluation with external limitations, data saturation is probably not reached as used in Grounded Theory, where the concept was developed. See context as described in (Max 8 participants in program) RESULTS --- *Feasibility data, Aim 1, quantitative and qualitative findings* Instead the findings is

		related to the aspect of "Information Power" as formulated by Malterud et al (2026). See end of "Discussion" for argumentation.
23. Transcripts returned	Were transcripts returned to participants for comment and/or correction?	No
**Domain 3: analysis and findings**		
Data analysis		
24. Number of data coders	How many data coders coded the data?	Three (EAL, CLN and MJ)
25. Description of the coding tree	Did authors provide a description of the coding tree?	Yes Table 3
26. Derivation of themes	Were themes identified in advance or derived from the data?	In advance in deductive analysis (Feasibility aspects) Inductively for aspect of program participation experience. Described in Heading: MATERIAL AND METHODS – Data analysis
27. Software	What software, if applicable, was used to manage the data?	Not applicable
28. Participant checking	Did participants provide feedback on the findings?	No, this was not planned
Reporting		
29. Quotations presented	Were participant quotations presented to illustrate the themes/findings? Was each quotation identified? e.g. participant number	Yes:<br>See result section
30. Data and findings consistent	Was there consistency between the data presented and the findings?	Yes
31. Clarity of major themes	Were major themes clearly presented in the findings?	Yes.<br>Table 3, and result section
32. Clarity of minor themes	Is there a description of diverse cases or discussion of minor themes?	Not applicable
