# Supplementary material for: Program evaluation of a wilderness experience for adolescents facing cancer: A time in nature to heal, connect and find strength
Source: PLoS One. 2023 Oct 3;18(10):e0291856. doi: 10.1371/journal.pone.0291856 (PMC10547176; doi:10.1371/journal.pone.0291856)
Supplement: S3 Appendix — (PDF) [file pone.0291856.s003.pdf]

SYATS – Thematic Interview guide for exit interview.

The interviews will be performed in an open manner where the participants will be asked to describe their experiences of the trip and how it has made them feel, further in depth about the wilderness setting and the leaders.

Interviewing in thematic areas give possibilities for follow up questions, such as, please describe this, or that more; what did you mean by saying… What do you think made you feel…

The thematic areas gives an overall direction, but dependent on the interviewee other turns may take off.

Examples of themes:

GENERAL COMMENTS

- General impressions of participation in the program
    - Please describe your overall experience of being part of this trip …
    - Please describe the best things of the trip…
    - Please describe the not so good parts of this trip…
- Your experience of the different activities
    - The hiking
        - Easy/strenuous
    - The camping
        - Able to sleep
        - Camp set-up and packing up?
        - Food prep?
        - Clothes and shoes /wetness/clean/dry
    - Please describe activities that were easy, challenging, hard, impossible
    - Please describe what supported you in taking part in the different activities
    - Please describe what held you back in participating in some activities (if any?)
    - Was any activity too hard?
    - What kind of feelings did you experience while taking part in the activities?
    - Please describe the most fun or rewarding activity
    - Please describe the least fun or negative activity

- The leaders/facilitators
    - Please describe how you perceived the leaders
    - In what way did you feel that they were able to support you on this trip?
    - Can you please give some tips on how they can be more supportive?
- The other participants-SOCIAL
        - Please describe in general how you feel about the other participants on the trip. Were you able to become friends with others?
            - Was it easy or hard?
        - In what way did you feel connected to them? (if at all)…
        - In what way did they respect and listen to you?
        - Positive aspects of the social experience?
        - Negative aspects of the social experience?
- The NATURE – the Three sisters wilderness

- Can you please describe a bit of your previous experience of being out in "the wild"
    - How do you feel about the nature here?
    - Was there any special place here where you felt more comfortable than others?
    - Was there any time that you were afraid of being out in the wild, if so when, and how were you able to handle it…
- Health aspects
    - Is there anything about your health that felt changed during the wilderness trip? (Please describe what and how)
- Harm/injury
    - Did you ever feel unsafe during the trip? (if so when, why, and how were you supported?)
    - Did you get hurt during the trip (if so when, why, and how were you supported?)
        - Blisters, infections, insects, ankle strains etc.
    - Did something "bad" happen, that you did not tell the leaders (what happened, and why not?)
- Reasons for signing up in the first place
    - Why choose to come on this trip?
    - What were you expectations?
    - Were your expectations met?
- Would you recommend this event to a friend of yours? (why or why not)
- Is there something more that you would like to add?
