# Supplementary material for: Program evaluation of a wilderness experience for adolescents facing cancer: A time in nature to heal, connect and find strength
Source: PLoS One. 2023 Oct 3;18(10):e0291856. doi: 10.1371/journal.pone.0291856 (PMC10547176; doi:10.1371/journal.pone.0291856)
Supplement: Attachment — Submitted filename: Response to reviewers PloSONE-SYATS May30 2023.pdf [file pone.0291856.s004.pdf]

RESPONSE TO REVIEWERS

Från: em.pone.0.829559.33c6fb1d@editorialmanager.com <em.pone.0.829559.33c6fb1d@editorialmanager.com> **För** PLOS ONE
Skickat: den 10 april 2023 15:24
Till: Jong, Mats <mats.jong@miun.se>
Ämne: PLOS ONE Decision: Revision required [PONE-D-22-28734] - [EMID:d63a924362e59548]

PONE-D-22-28734
Program evaluation of a wilderness experience for adolescents facing cancer: A time in nature to heal, connect and find strength
PLOS ONE

Dear Dr. Jong,

Thank you for submitting your manuscript to PLOS ONE. After careful consideration, we feel that it has merit but does not fully meet PLOS ONE's publication criteria as it currently stands. Therefore, we invite you to submit a revised version of the manuscript that addresses the points raised during the review process.

Please submit your revised manuscript by May 25 2023 11:59PM. If you will need more time than this to complete your revisions, please reply to this message or contact the journal office at plosone@plos.org. When you're ready to submit your revision, log on to https://www.editorialmanager.com/pone/ and select the 'Submissions Needing Revision' folder to locate your manuscript file.

Please include the following items when submitting your revised manuscript:

- A rebuttal letter that responds to each point raised by the academic editor and reviewer(s). You should upload this letter as a separate file labeled 'Response to Reviewers'.
- A marked-up copy of your manuscript that highlights changes made to the original version. You should upload this as a separate file labeled 'Revised Manuscript with Track Changes'.
- An unmarked version of your revised paper without tracked changes. You should upload this as a separate file labeled 'Manuscript'.

If you would like to make changes to your financial disclosure, please include your updated statement in your cover letter. Guidelines for resubmitting your figure files are available below the reviewer comments at the end of this letter.

If applicable, we recommend that you deposit your laboratory protocols in protocols.io to enhance the reproducibility of your results. Protocols.io assigns your protocol its own identifier (DOI) so that it can be cited independently in the future. For instructions see: https://journals.plos.org/plosone/s/submission-guidelines#loc-laboratory-protocols. Additionally, PLOS ONE offers an option for publishing peer-reviewed Lab Protocol articles, which describe protocols hosted on protocols.io. Read more information on

sharing protocols at https://plos.org/protocols?utm_medium=editorial-email&utm_source=authorletters&utm_campaign=protocols.

We look forward to receiving your revised manuscript.

Kind regards,

Cho Lee Wong, PhD
Academic Editor
PLOS ONE

Journal Requirements:

When submitting your revision, we need you to address these additional requirements.

1. Please ensure that your manuscript meets PLOS ONE's style requirements, including those for file naming. The PLOS ONE style templates can be found at https://journals.plos.org/plosone/s/file?id=wjVg/PLOSOne_formatting_sample_main_body.pdf and https://journals.plos.org/plosone/s/file?id=ba62/PLOSOne_formatting_sample_title_authors_affiliations.pdf

2. Please include a complete copy of PLOS' questionnaire on inclusivity in global research in your revised manuscript. Our policy for research in this area aims to improve transparency in the reporting of research performed outside of researchers' own country or community. The policy applies to researchers who have travelled to a different country to conduct research, research with Indigenous populations or their lands, and research on cultural artefacts. The questionnaire can also be requested at the journal's discretion for any other submissions, even if these conditions are not met. Please find more information on the policy and a link to download a blank copy of the questionnaire here: https://journals.plos.org/plosone/s/best-practices-in-research-reporting. Please upload a completed version of your questionnaire as Supporting Information when you resubmit your manuscript.

It is filled out and included as an attachment with the resubmission.

3. Please provide additional details regarding participant consent. In the ethics statement in the Methods and online submission information, please ensure that you have specified what type you obtained (for instance, written or verbal, and if verbal, how it was documented and witnessed). If your study included minors, state whether you obtained consent from parents or guardians. If the need for consent was waived by the ethics committee, please include this information.

In doing research with vulnerable participants, especially children and adolescents with cancer it is crucial that we adopt highly ethical methods, which we think we employed. In section 2.4, lines 182-184 of our submitted version we believe we addressed this

issue, but we added one additional phrase for clarity (underlined): "Written informed consent for participation in the evaluation was obtained from guardians of those younger than 18, and for those age 18 and older. In addition, the study obtained written assent from minors for participation in the study (Supplement 2)."

4. Thank you for stating in your Funding Statement:
"HRO – Funding for the See You at the Summit program was provided in part by the OHSU Knight Cancer Institute Community Partnership Program.
URL: https://www.ohsu.edu/knight-cancer-institute/community-partnership-program-grants
EAL - Funding for the Evaluation was provided by the University of California San Francisco, Osher Center for Integrative Medicine, Integrative Oncology Research Pilot Award. URL: https://oshercenter.org/research/funding-opportunities/"

Please provide an amended statement that declares *all* the funding or sources of support (whether external or internal to your organization) received during this study, as detailed online in our guide for authors at http://journals.plos.org/plosone/s/submit-now. Please also include the statement "There was no additional external funding received for this study." in your updated Funding Statement.
Please include your amended Funding Statement within your cover letter. We will change the online submission form on your behalf.

See below under point #5.

5. Thank you for stating the following financial disclosure:
"HRO – Funding for the See You at the Summit program was provided in part by the OHSU Knight Cancer Institute Community Partnership Program.
URL: https://www.ohsu.edu/knight-cancer-institute/community-partnership-program-grants
EAL - Funding for the Evaluation was provided by the University of California San Francisco, Osher Center for Integrative Medicine, Integrative Oncology Research Pilot Award. URL: https://oshercenter.org/research/funding-opportunities/"

Please state what role the funders took in the study. If the funders had no role, please state: ""The funders had no role in study design, data collection and analysis, decision to publish, or preparation of the manuscript.""
If this statement is not correct you must amend it as needed.
Please include this amended Role of Funder statement in your cover letter; we will change the online submission form on your behalf.

Thank you for helping us to clarify the above. We have added relevant content related to funders in our cover letter. Thank you for making the relevant changes online.

6. Thank you for stating the following in the Competing Interests section:
"EAL, CLO, MCJ and MJ: The authors have declared that no competing interests exist.

HRO: I have read the journal's policy and the authors of this manuscript have the following competing interests: Founder and director of See You At The Summit, the program that has been evaluated. As co-author I have been involved in the full process of the program evaluation, but the full group of authors has together made sure that reporting of results has been done openly and unbiased."

Please confirm that this does not alter your adherence to all PLOS ONE policies on sharing data and materials, by including the following statement: ""This does not alter our adherence to PLOS ONE policies on sharing data and materials." (as detailed online in our guide for authors http://journals.plos.org/plosone/s/competing-interests). If there are restrictions on sharing of data and/or materials, please state these. Please note that we cannot proceed with consideration of your article until this information has been declared.

Please include your updated Competing Interests statement in your cover letter; we will change the online submission form on your behalf.

This content has been added to the statement of competing interests in our cover letter. Thank you for making the change online.

7. In your Data Availability statement, you have not specified where the minimal data set underlying the results described in your manuscript can be found. PLOS defines a study's minimal data set as the underlying data used to reach the conclusions drawn in the manuscript and any additional data required to replicate the reported study findings in their entirety. All PLOS journals require that the minimal data set be made fully available. For more information about our data policy, please see http://journals.plos.org/plosone/s/data-availability.

Upon re-submitting your revised manuscript, please upload your study's minimal underlying data set as either Supporting Information files or to a stable, public repository and include the relevant URLs, DOIs, or accession numbers within your revised cover letter. For a list of acceptable repositories, please see http://journals.plos.org/plosone/s/data-availability#loc-recommended-repositories. Any potentially identifying patient information must be fully anonymized.

We have contacted PlosONE and received an exception on the submission of the dataset to a public repository due to the small number and the extensive photo and videographic documentation of the trip, making it easier to identify participants. We have however a clean data set that we will maintain and that can be used by qualified researchers. We have outlined the process for access to the data in the Cover letter to the editor. As well as in the appropriate section of the online submission system

Additionally, please revise text regarding qualitative data as follows:

"Data from observation and qualitative interviews contains sensitive information and is available upon request from the medical director, Dr Joshua Hurwitz joshua.j.hurwitz@gmail.com, and

after approval from the Internal Review Board Ethics Committee For Legacy Health (Portland OR, USA)"

Important: If there are ethical or legal restrictions to sharing your data publicly, please explain these restrictions in detail. Please see our guidelines for more information on what we consider unacceptable restrictions to publicly sharing data: http://journals.plos.org/plosone/s/data-availability#loc-unacceptable-data-access-restrictions. Note that it is not acceptable for the authors to be the sole named individuals responsible for ensuring data access.

See information about access to data in the cover letter.

We will update your Data Availability statement to reflect the information you provide in your cover letter.

8. Please include captions for your Supporting Information files at the end of your manuscript, and update any in-text citations to match accordingly. Please see our Supporting Information guidelines for more information: http://journals.plos.org/plosone/s/supporting-information.

Supporting information files have been updated and in-text citations have been updated to match accordingly.

9. Please review your reference list to ensure that it is complete and correct. If you have cited papers that have been retracted, please include the rationale for doing so in the manuscript text, or remove these references and replace them with relevant current references. Any changes to the reference list should be mentioned in the rebuttal letter that accompanies your revised manuscript. If you need to cite a retracted article, indicate the article's retracted status in the References list and also include a citation and full reference for the retraction notice.

All our citations are valid and no changes has been made since previous version.

Additional Editor Comments:

Dear Authors

Thanks for the opportunity to review this manuscript. The topic of this manuscript is interesting and fills a gap where few services or research projects have examined adolescents' use of nature and wilderness experiences. I just have a few small comments for your consideration:
1) Line 83: Please specify the type of chronic disease.

We provide additional information for the reviewer below to this important and common issue of long-term late effects to the health of young cancer survivors. In the effort to contain the

length we have added a single sentence to the manuscript to provide some more information, underlined below.

Line 84: "At least 66% of child and adolescent cancer survivors have one or more chronic diseases as defined by the Common Terminology Criteria for Adverse Events (version 3) (39) including conditions such as congestive heart failure, major joint replacement, second malignant neoplasms, severe cognitive dysfunction, coronary artery disease, or renal failure.(11)"

For the reviewers: Data was collected from 10,397 long-term cancer survivors and 3034 of their siblings on chronic health problems including congestive heart failure, major joint replacement, second malignant neoplasms, severe cognitive dysfunction, coronary artery disease, cerebrovascular accident, renal failure or dialysis, uncorrectable hearing loss, loss of vision and ovarian failure.

The severity of the conditions was scored using the Common Terminology Criteria for Adverse Events (version 3). This is a scoring system developed through the National Cancer Institute by a multidisciplinary group and is widely used to score both acute and chronic health conditions in patients with cancer and survivors of all ages. (Cancer Therapy Evaluation Program, Common terminology criteria for adverse events, version 3.0.) The system grades conditions as mild (grade 1), moderate (grade 2), severe (grade 3), life-threatening or disabling (grade 4), or fatal (grade 5). For our study, a total of 137 health conditions were scored.

2) Line 93: Please elaborate on previous programme that addressed psychosocial challenges and explain why wilderness therapy was chosen.

We have now included additional information on existing psychosocially oriented programs and how a nature-based intervention under study fits into this history. Also, see the following section 1.3 where we justify in detail why we are testing a nature-based therapy.

Lines 99-105 "Due to a host of psychosocial stresses following cancer, psychosocial guidelines recommend providing psychosocial interventions to survivors.(29) A wide variety of interventions exist to address long term physical and emotional sequelae of cancer including educational(30) cognitive behavioral, (31) physical exercise(32-34), health behavior change(35) and social support programs. A number of nature-based programs exist, such as cancer camps and adventure therapy (usually for young adult cancer survivors) and are reviewed here.(36) Few programs explicitly evaluate the role of nature in healing.

3) Line 147: Why money situation evaluated in the baseline?

Psychosocial interventions have not always been accessible to all patients regardless of socioeconomic status (SES). This program made it a priority to provide all aspects of the program for free to remove SES barriers.

4) Line 153: It is suggested that Table 2 be placed in the supplementary file rather than the main text.

Good idea. It is now labeled Appendix, Table 2.

5) Line 292: It is recommended to present the contents of Table 3 in text.

Since we put Table 2 in the Appendix, the Recruitment and Tracking Table is now Table 2 (not Table 3). Typically, one does not put into text content that can be read from the table but in deference to the reviewer we now describe Table 2 results. Here is the edited text from Section 3.1, Lines 302-308.

Lines 326-334 (new lines are underlined) "The SYATS <u>recruitment goal was</u> eight participants (the Forest Service permit maximum group limit is 12, <u>which includes 8 participants and 4 staff members</u>. Hospital staff (nurses, social workers, and ambassadors) approached teens/families and introduced the wilderness program. A total of 21 teenagers, aged 12-17 were referred to, or expressed interest in participating in SYATS. Among this group, the cancer diagnoses included: brain tumor, leukemia, germ cell tumor, and lymphoma among others. <u>Among the 21 with interest in SYATS, 4 (19%) were ultimately not eligible, 6 (35%) did not contact SYATS again, 3 (18%) were not interested or had a scheduling conflict and 8 (47%) signed up (See Table 2).</u> Retention was 100% with all eight teens participating from day 1-9."

6) Line 384: Table 5, please add the actual mean score along with range

In (now) Table 4, we have added the mean scores and range of mean scores for baseline, day 9 FU, and 3-mo FU. We also still include the differences between baseline and the two follow-ups time-points.

7) In the discussion, suggest to compare the content and results of the current wilderness programme with previous programme.

We now add the following information.

Lines 674-677. "The SYATS program provides preliminary data that is in accordance with data from other outdoor or adventure therapy programs including increased social involvement, increased self-esteem, self-confidence, self-efficacy, social support, and physical activity. (35)"

As described on page 29, previous programs are substantially different. They were not aimed at this age group, for this timespan, nor did they include an evaluation of the role of nature as well as mindful/reflective activities (journaling, meditation, yoga, meditation). None used the strongly validated PROMIS measures. See Scoping review referenced above (Jong, 2021).

[Note: HTML markup is below. Please do not edit.]

Reviewers' comments:

Reviewer's Responses to Questions

Comments to the Author

1. Is the manuscript technically sound, and do the data support the conclusions?

The manuscript must describe a technically sound piece of scientific research with data that supports the conclusions. Experiments must have been conducted rigorously, with appropriate controls, replication, and sample sizes. The conclusions must be drawn appropriately based on the data presented.

Reviewer #1: Yes

Reviewer #2: Yes

Reviewer #3: Yes

Reviewer #4: Yes

Reviewer #5: Yes

---

2. Has the statistical analysis been performed appropriately and rigorously?

Reviewer #1: Yes

Reviewer #2: Yes

Reviewer #3: Yes

Reviewer #4: Yes

Reviewer #5: N/A

---

3. Have the authors made all data underlying the findings in their manuscript fully available?

The PLOS Data policy requires authors to make all data underlying the findings described in their manuscript fully available without restriction, with rare exception (please refer to the Data Availability Statement in the manuscript PDF file). The data should be provided as part of the manuscript or its supporting information, or deposited to a public repository. For example, in addition to summary statistics, the data points behind means, medians and variance measures should be available. If there are restrictions on publicly sharing data—e.g. participant privacy or use of data from a third party—those must be specified.

Reviewer #1: Yes

Reviewer #2: No

Reviewer #3: Yes

Reviewer #4: Yes

Reviewer #5: No

In the resubmission we have described how data can be accessed after contacting the SYATS Medical director

---

4. Is the manuscript presented in an intelligible fashion and written in standard English?

PLOS ONE does not copyedit accepted manuscripts, so the language in submitted articles must be clear, correct, and unambiguous. Any typographical or grammatical errors should be corrected at revision, so please note any specific errors here.

Reviewer #1: Yes

Reviewer #2: Yes

Reviewer #3: Yes

Reviewer #4: Yes

Reviewer #5: Yes

---

5. Review Comments to the Author

Please use the space provided to explain your answers to the questions above. You may also include additional comments for the author, including concerns about dual publication, research ethics, or publication ethics. (Please upload your review as an attachment if it exceeds 20,000 characters)

Reviewer #1: 1-The authors should explain about how to screen the qualitative data and should show the data that related or not related between participants.
2-In discussion part the authors should clearly describe why SYATS program that effect in participant.

In the results, we have tried to exemplify the existing variety between (and sometimes within) individuals within quotes regarding feasibility, but also in terms of representation in themes and categories.

Reviewer
#2: Overall, a strong piece of writing that might use some tweaking in terms of flow and presentation.

The authors may also provide research implications.

Overall, the writing is strong, but the flow and presentation could be improved.

As we have edited the article we worked to improve the flow and presentation by removing redundancy, editing syntax, and shortening.

The authors may also provide research implications.

+Reviewer #3: Program evaluation of a wilderness experience for adolescents facing cancer: A time in nature to heal, connect and find strength. This manuscript good presentation, Overall, this paper has clear research issues, appropriate study methods, and presents interesting results.

Thank you!

+Reviewer #4: Program evaluation of a wilderness experience for adolescents facing cancer: A time in nature to heal, connect and find strength. Overall clear, concise and interest to reader. Its interesting for publish jouraal. Accept

Thank you.

Reviewer #5: The authors have provided a detailed description of a program of a wilderness experience for adolescents with cancer. the intent is noble and good- and the program is described discursively in a study of 8 participants, which really constitutes a preliminary/feasibility study.

We agree with the reviewer comment that the study is a preliminary/feasibility study and we hope we made that clear throughout where we mentioned this in the abstract, methods and discussion sections.

The small number of subjects is not really analyzed- nor evaluated in a systematic way beyond the success of participation. the manuscript describes the success of the project and provides ample- perhaps too many detailed quotations. the manuscript could benefit greatly from substantial shortening- and moving many of the anecdotal quotes to a series of supplementals documents organized by the questions asked- and the responses. as presented, it is repetitive and works against its purpose- a demonstration of the feasibility and utility of a program- that could be extended in scope.

We have now moved material that might be redundant into the Appendix Under Section 3. We originally chose to organize the material around a list of criteria often used to assess pilot studies. (National Center for Complementary and Integrative Health. Pilot Studies: Common Uses and Misuses. Framework for Developing and Testing Mind and Body Interventions 2019 Washington, DC: NCCIH; 2019). We outlined these criteria at the end of the introduction (lines 130-135). We still think it makes sense to organize the material this way as it reflects that the evaluation criteria were thoroughly addressed. It also reflects the way we outlined the structure of the manuscript at the end of the introduction section where we say "The primary aim of the study is to describe the feasibility, acceptability, and safety of See You at the Summit (SYATS) wilderness program. The secondary aim describes the impact on participants including a quantitative assessment of global and mental health as well as qualitative findings and observations describing the program participation experience of SYATS participants. Starting on page 21, Line 472 we organized the qualitative analysis around themes that emerged which is a fairly standard way to report qualitative findings. The themes are described in Table 5.

the numbers are too small to establish any rigorous statistical analyses.

We are in full agreement with the reviewer. The number of participants (while meeting our enrollment goal) was too small to draw any statistically significant conclusions about effectiveness. This is intended as a pilot study allowing the SYATS team to work out the details of the intervention delivery and evaluation components. Any statistical work will help us to understand trends and help us to calculate sample sizes for future studies, but cannot determine effectiveness. We hope we made that clear in a number of places in the manuscript (in the methods, results and discussion sections). I include that material below to show that the authors fully acknowledge the limitations of the small sample.

Methods: Lines 151-153. We say "This data should be used for future sample size calculations and for descriptive purposes only given the small sample size."

Results: Line 456. "While the small sample size precluded meaningful significance testing, PROMIS baseline data showed ….. As can be the case with small numbers, the mean changes can be strongly influenced by a few persons."

Discussion, Line 701 "As expected, small numbers limit conclusions about the program impact,…" and Lines 770-776 "However, in this small group, we caution against over interpretation of mean PROMIS sub-scale scores since a few people can alter the group mean. Use of multimethod assessments provided rich data to better understand where participants stand in relation to norms (PROMIS) and to understand the lived experience."

The final sentence of the abstract offers a vague statement that should be reworded. "controlled" studies is confusing and should not imply a randomization or standard

clinical study for a handful of outcomes. Please be careful here and consider the next steps- is it continue the program or is it to develop clear and statistically compelling design- which would be very hard given the heterogeneity of patients, treatment schedules and timing of the structured experiences.

Related to the final statement of the conclusion in the abstract—we have edited that statement to clarify our meaning. It now reads (Line 51) "Larger randomized controlled studies are needed to learn whether these programs can improve psychosocial outcomes." We understand why you might say this and recognize the challenges in using the gold standard RCT study design in situations where it is difficult to blind participants and staff. That said, we do want to note that several co-authors have been involved in another similar wilderness intervention using a randomized controlled trial design that involves a wilderness adventure vs. a relaxing hotel spa retreat. Yes, you are correct, it is challenging, but this new trial was funded, and the intervention has been delivered with success. (Manuscript is in progress). The protocol reference is listed here.

Jong MC, Mulder E, Kristoffersen AE, et al. Protocol of a mixed-method randomised controlled pilot study evaluating a wilderness programme for adolescent and young adult cancer survivors: the WAYA study. BMJ Open 2022;12:e061502. doi:10.1136/ bmjopen-2022-061502

---

6. PLOS authors have the option to publish the peer review history of their article (what does this mean?). If published, this will include your full peer review and any attached files.

If you choose "no", your identity will remain anonymous but your review may still be made public.

Do you want your identity to be public for this peer review? For information about this choice, including consent withdrawal, please see our Privacy Policy.

Reviewer #1: No

Reviewer #2: No

Reviewer #3: No

Reviewer #4: No

Reviewer #5: No

[NOTE: If reviewer comments were submitted as an attachment file, they will be attached to this email and accessible via the submission site. Please log into your account, locate the manuscript record, and check for the action link "View Attachments". If this link does not appear, there are no attachment files.]

While revising your submission, please upload your figure files to the Preflight Analysis and Conversion Engine (PACE) digital diagnostic tool, https://pacev2.apexcovantage.com/. PACE helps ensure that figures meet PLOS requirements. To use PACE, you must first register as a user. Registration is free. Then, login and navigate to the UPLOAD tab, where you will find detailed instructions on how to use the tool. If you encounter any issues or have any questions when using PACE, please email PLOS at figures@plos.org. Please note that Supporting Information files do not need this step.
